# A Broad-Based Characterization of a Cell-Penetrating, Single Domain Camelid Bi-Specific Antibody Monomer That Targets STAT3 and KRAS Dependent Cancers

**DOI:** 10.3390/ijms23147565

**Published:** 2022-07-08

**Authors:** Sunanda Singh, Genoveva Murillo, Justin Richner, Samara P. Singh, Erica Berleth, Vijay Kumar, Rajendra Mehta, Vijay Ramiya, Ashutosh S. Parihar

**Affiliations:** 1 Singh Biotechnology, 1547 Fox Grape Loop, Lutz, FL 33558, USA; vramiya70@gmail.com; 2 IIT Research Institute, 10 W. 35th Street, Chicago, IL 60616, USA; gmurillo@iitri.org (G.M.); rajumehta47@gmail.com (R.M.); 3 Department of Microbiology & Immunology, University of Illinois Chicago, E829 Medical Sciences Building, Chicago, IL 60612, USA; richner@uic.edu; 4 Division of Surgical Oncology, Department of Surgery, University of Miami Miller School of Medicine, Miami, FL 33136, USA; sxs1618@miami.edu; 5 Acudex, Inc., 701 Ellicott Street, CBLS, Buffalo, NY 14203, USA; berleth@aesku.com (E.B.); vjkumar45@gmail.com (V.K.)

**Keywords:** KRAS, STAT3, bi-specific, VHH, PD-L1, VEGF, blood brain barrier

## Abstract

STAT3 and KRAS regulate cell proliferation, survival, apoptosis, cell migration, and angiogenesis. Aberrant expression of STAT3 and mutant active forms of KRAS have been well-established in the induction and maintenance of multiple cancers. STAT3 and KRAS mutant proteins have been considered anti-cancer targets; however, they are also considered to be clinically “undruggable” intracellular molecules, except for KRAS(G12C). Here we report a first-in-class molecule, a novel, single domain camelid VHH antibody (15 kDa), SBT-100, that binds to both STAT3 and KRAS and can penetrate the tumor cell membrane, and significantly inhibit cancer cell growth. Additionally, SBT-100 inhibits KRAS GTPase activity and downstream phosphorylation of ERK in vitro. In addition, SBT-100 inhibits the growth of multiple human cancers in vitro and in vivo. These results demonstrate the feasibility of targeting hard-to-reach aberrant intracellular transcription factors and signaling proteins simultaneously with one VHH to improve cancer therapies.

## 1. Introduction

Signal transducer and activator of transcription 3 (STAT3) is both a signaling molecule and a transcription factor, while the mammalian homolog of Kirsten RAS (KRAS) is a signal relaying GTP-binding protein, however, both have been popular yet elusive targets in the world of cancer therapy. Traditional chemotherapies alone are often insufficient and can result in significant toxicities, rendering many human malignancies difficult to treat, including pancreatic cancers, triple-negative breast cancers (TNBC), glioblastomas, and sarcomas [1,2,3]. In addition, new targeted therapies can often become ineffective eventually, due to the development of drug resistance in cancer cells, often linked to the blockade of key signaling pathways [4]. Oncogenic mutations, loss of tumor suppressor genes, overexpression of normal proteins, or some combination of these events also contribute to drug resistance [5,6,7]. Janus kinase (JAK)/STAT pathway is a key modulator of cellular growth, differentiation, and inflammatory response [7,8,9]. Elevated phosphorylated STAT3 (pSTAT3) was associated with a poor prognosis of solid tumors [10]. Activated STAT3 forms homodimers and translocate to the nucleus, where it binds DNA to initiate the transcription of target genes associated with cellular growth, proliferation, anti-apoptosis, angiogenesis, immunosuppression, and invasion/migration [9]. Due to its central role in tumor processes, STAT3 has been considered a potential anticancer target since its first description as an oncogene in 1998 and has led to the evaluation of STAT3 inhibitors for their antitumor activity in vitro and in vivo using experimental tumor models [11]. However, most of these inhibitors are yet to be translated to clinical use for cancer treatment, primarily due to pharmacokinetics, efficacy, and safety obstacles. KRAS mutations have been shown to be the driver mutation for ~25% of human cancers, while most frequently present in pancreatic (98%) and colorectal (53%) cancers [12]. The mutant form retains GTP without hydrolyzing it, thereby becoming constitutively active. Many have focused on developing small molecule targets for the KRAS mutant for decades. However, problems in detecting binding pockets for these small molecules to bind KRAS have rendered this a nearly impossible task, and thus far only an anti-KRAS(G12C) inhibitory small molecule drug has been approved. Recently, cyclic peptides binding to a shallow cleft near the Switch II region comprising of two alpha helices and a central beta strand have been described [13].

Cancer cells utilize pSTAT3 as an escape mechanism to become resistant to chemotherapy and radiation therapy [14,15]. Inhibiting STAT3 with small molecule inhibitors not only suppresses cancer growth, activates apoptosis, and inhibits angiogenesis, but it also has been shown to remodel the stroma of pancreatic cancers [16]. Inhibiting STAT3 in human patients during Phase 1 studies has demonstrated this to be a safe and well-tolerated approach [17]. Studies have focused on identifying novel small molecule inhibitors of STAT3, which act either by inhibiting phosphorylation of STAT3, DNA binding, or the formation of functional STAT3 dimers. However, direct usage of a cell penetrating anti-STAT3 antibody has not been successful due to size limited difficulties in cellular penetration of the antibodies [18]. An earlier dogma has forwarded the idea that nanobodies (VHH) do not freely cross cell membranes [19].

A significant fraction, about 40%, of camelid antibodies is comprised of only two heavy chain immunoglobulins with one variable domain (VHH) per heavy chain. Camelid VHHs have been used to target multiple extracellular targets (e.g., IL-6 receptor, IL-17, TNF-α, VWF, and others), and several VHHs are in various stages of human clinical trials (Phases II and III) [19], with no major side effects or toxicity reported. One VHH, Caplacizumab, has been approved by FDA and has been successfully commercialized for treating adult-acquired thrombotic thrombocytopenic purpura [20]. In this report, we describe our VHH SBT-100′s capabilities to penetrate cell membranes and bind to STAT3; additionally, it has the ability to cross-react with KRAS (mutated and unmutated forms), acting as a bi-specific antibody, with nM binding affinity. We further provide evidence for the utility of SBT-100 as an anti-cancer drug by showing that SBT-100: (1) crosses the cell membrane and binds to intracellular STAT3, (2) inhibits phosphorylation of STAT3, (3) decreases total STAT3, (4) blocks IL-6 mediated translocation of activated STAT3 into the nucleus, which prevents pSTAT3 dimers from binding to its target genes, (5) inhibits expression of VEGF, (6) inhibits cell surface expression of checkpoint inhibitor PD-L1 on the tumor cell surface, (7) inhibits KRAS-GTPase activity and downstream ERK phosphorylation, (8) exhibit wide-ranging anti-tumor cell growth in vitro, and (9) induce tumor suppression in athymic xenograft mouse models for triple-negative breast cancer cell line with KRAS(G13D) mutation (MDA-MB-231) and a pancreatic cancer cell line with KRAS(G12D) mutation (PANC-1), without any observable toxicity. Additionally, the biological effects of SBT-100 last between 72 h in vitro and 7 days in vivo. To the best of our knowledge, no other group has described an unmodified cell penetrating VHH that gives a therapeutic effect in cancer. SBT-100 is a novel VHH that penetrates the cell membrane to give a therapeutic effect against human cancers, thus representing a significant clinical potential. This unique property of SBT-100 and the mechanism of action of cell penetration is under investigation.

## 2. Results

### 2.1. Binding Characteristics of Anti-STAT3 and Anti-KRAS Camelid VHH: SBT-100

To first create an anti-STAT3 camelid antibody, a camel (*Camelus bactrianus*) was used for immunization with the recombinant human STAT3. After the immunization protocol was completed, peripheral blood cells were collected for total RNA isolation and two rounds of nested PCRs to create a VHH library. Next, in clones that were selected in this present study, we observed high-affinity binding of SBT-100 to recombinant STAT3 and KRAS, both wild type and mutated form (G12D), using Biacore 3000 (Table 1). For scouting, the sample was allowed to flow over the chip, and the binding of sample to the ligand was monitored in real-time. The affinity constant is expressed as the equilibrium dissociation constant (K_D_) which was determined as a ratio of dissociation and association rates. SBT-100 binds wild type KRAS with a K_D_ = 4.20 × 10^−9^, KRAS(G12D) with K_D_ = 1.50 × 10^−8^, and STAT3 with K_D_ = 2.24 × 10^−8^. SBT-100 does not bind an irrelevant antigen (12-Lipoxygenase). Although STAT3 and KRAS molecules are highly conserved in nature, they do not share significant homology in their protein sequences. Unlike conventional monoclonal antibodies which mostly recognize flat and convex antigenic surfaces, the CDR3 loop of VHHs is longer than conventional VHHs, which allows its elongated paratope binding to non-conventional epitopes such as concave-shaped protein clefts such as enzyme active sites [21]. While binding to its immunogen STAT3 was expected, the cross-reactivity to non-homologous KRAS was not, especially with a higher affinity than STAT3 immunogen itself. Although structural studies on binding have yet to be carried out to explain this impressive heteroclitic cross-reactivity, it is theoretically feasible that SBT-100 binds to a shallow cleft described for KRAS by electrostatic interactions [13], or SBT-100 may interact with a yet unidentified pocket in KRAS. Interestingly, as shown in Table 1, anti-KRAS VHH (SBT-102) did not cross-react with STAT3 protein.

### 2.2. Cellular Penetration by SBT-100

**Figure 1:**
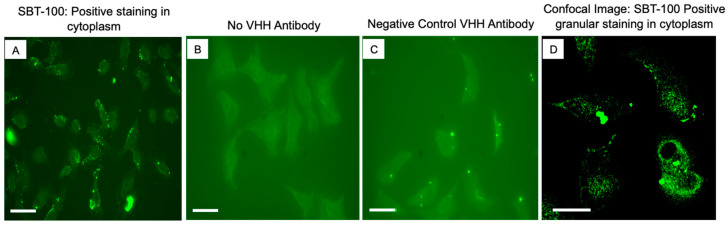
SBT-100 penetrates cell membrane of TNBC cell line, MDA-MD-231. (**A**) Immunofluorescence staining of MDA-MB-231 cells incubated with SBT-100 antibody showed positive cytoplasmic staining using the anti-His tag antibody in green channel. (**B**) MDA-MB-231 cells with no VHH antibody were negative. (**C**) No cytoplasmic positive staining was seen with the negative control irrelevant VHH antibody (anti-HIV 1-reverse transcriptase VHH) with traditional fluorescence microscopy. (**D**) Confocal image of SBT-100 detection is shown as granular staining located throughout the cytoplasm. The observed staining of SBT-100 in (**A**,**D**) is cytosolic and not endocytic. The scale bars in the lower-left corner of each panel represent 100 µm.

**Figure 2:**
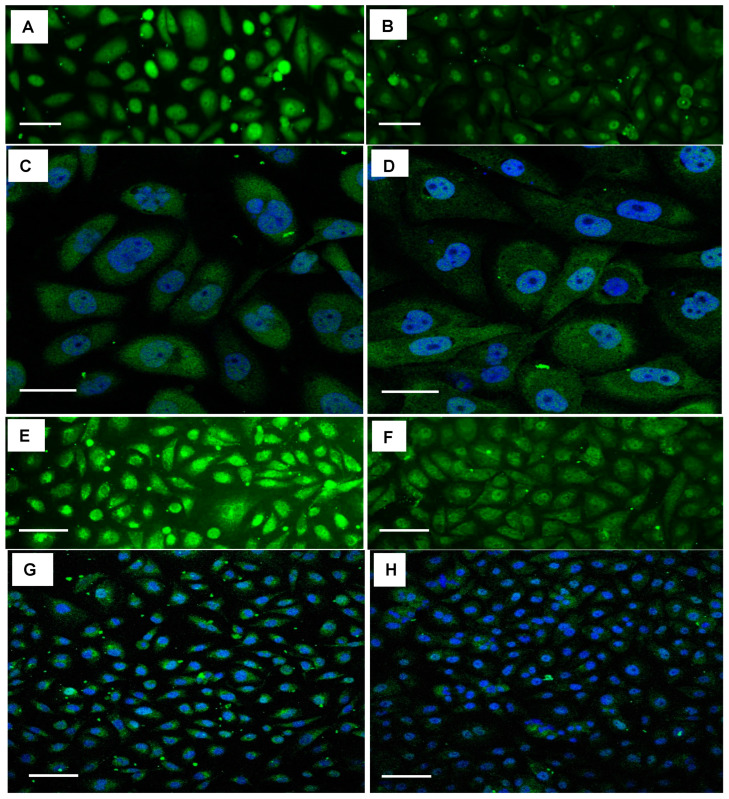
SBT-100 reduces intracellular levels of both phosphorylated and total STAT3. Confocal fluorescent microscopy demonstrates SBT-100 decreases intracellular levels of total (t) STAT3 in MDA-MB-231 cells, with the nuclei detection with DAPI. Fluorescent images of tSTAT3 in MDA-MB-231 cells, either untreated (**A**) or following 6 h of SBT-100 treatment (150 μg/mL) (**B**). Confocal images of tSTAT3 in MDA-MB-231 cells, either untreated (**C**) or following 6 h of SBT-100 treatment (150 μg/mL) (**D**), both with the nuclei detection with DAPI. Confocal fluorescent microscopy demonstrates SBT-100 decreases intracellular levels of phosphorylated (p) STAT3 in MDA-MB-231 cells, with the nuclei detection with DAPI. Fluorescent images of pSTAT3 in MDA-MB-231 cells, either untreated (**E**) or following 6 h of SBT-100 treatment (150 μg/mL) (**F**). Confocal images of pSTAT3 in MDA-MB-231 cells, either untreated (**G**) or following 6 h of SBT-100 treatment (150 μg/mL) (**H**), both with the nuclei detection with DAPI. The scale bars in the lower-left corner of each panel represent 100 µm.

We next determined the ability of SBT-100 VHH to penetrate the cell membrane and bind intracellular STAT3 using immunofluorescence analyses (IFA) of MDA-MD-231, a TNBC cell line. As shown in Figure 1A, cells treated with SBT-100 exhibited intracellular localization of SBT-100 as well as some membrane association as shown using the anti-GST tag antibody. As a negative control, MDA-MB-231 cells were cultured with an anti-HIV 1 reverse transcriptase (RT) VHH ((Figure 1C). Anti-HIV 1-RT VHH was developed in-house; because the MDA-MB-231 cells do not produce HIV 1 viruses; no intracellular staining was noted in these cells. As expected, the cells treated with vehicle showed no staining (Figure 1B). Confocal fluorescent microscopy revealed that SBT-100 is present as granular staining dispersed throughout the cytoplasm of MDA-MB-231 cells following treatment with SBT-100 (Figure 1D). Next, we determined the effect of SBT-100 on intracellular levels of STAT3. As shown in Figure 2A,B, IFA indicated that SBT-100 decreased intracellular levels of total (t) STAT3. Furthermore, there was a redistribution of tSTAT3 from predominantly nuclear and around the nucleus to tSTAT3 dispersed throughout the cell, indicating that nuclear localization of tSTAT3 is reduced in SBT-100-treated MDA-MB-231 cells. Confocal fluorescent image analysis for both tSTAT3 and DAPI (4′,6-diamidino-2-phenylindole)-stained nuclei further supports this SBT-100-induced redistribution of tSTAT3 staining from nuclear/cell-centralized to staining throughout the whole cell cytoplasm (Figure 2C,D). IFA studies demonstrated the MDA-MB-231 cells incubated with SBT-100 demonstrated decreased pSTAT3 levels within 6 h. Figure 2E–H shows reduced levels of pSTAT3 after treatment, and that SBT-100 induced changes in pSTAT3 cellular localization mirrored those of tSTAT3. These data suggest that SBT-100 hinders the translocation of both total and phosphorylated STAT3 from the cytoplasm to the nucleus. Thus, both conventional and confocal (Figure 2E–H) fluorescent images revealed that SBT-100 decreased intracellular levels of pSTAT3 in MDA-MB-231 cells.

Bruce et al. [22] suggested poly cationic resurfacing of VHH antibodies in order to provide a net charge of +14 or +15 for rendering cell penetrability. However, our unmodified protein sequence analysis using the online tool “Prot Pi” (https://www.protpi.ch/Calculator/ProteinTool#Results (accessed on 17 July 2020)) provided a theoretical net charge of +2.3 at physiological pH, deviating substantially from the suggested optimal net charge of for resurfaced nanobodies with cations as +14. Thus, SBT-100 appears unique in its ability to penetrate the cell, bind non-homologous proteins (STAT3 and KRAS), and cross the blood-brain barrier (BBB) (described later). Others have shown cell penetration by VHH following cationic resurfacing of the membrane molecules; however, SBT-100′s ability to penetrate the cell membrane does not involve poly-cationic resurfacing or genetic engineering, or conjugation of SBT-100. As of this reporting, we are only speculating the mechanism of cell penetration by SBT-100 based on available literature data and are not aware of the fraction of SBT-100 entering into the cytosol. Please see the discussion section for additional information.

### 2.3. Inhibition of Phosphorylation and Nuclear Translocation of STAT3 with Subsequent Reduction in Checkpoint Molecules

**Figure 3:**
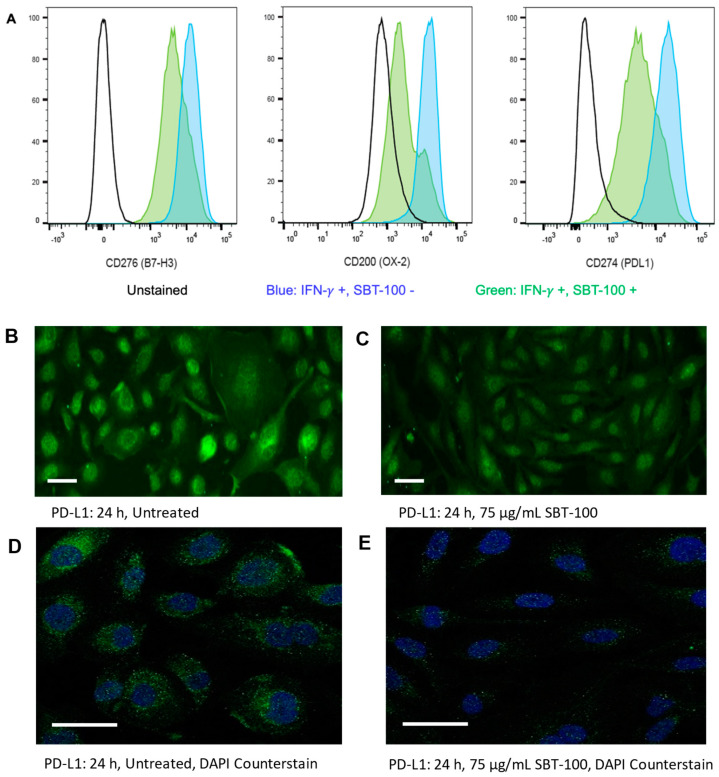
SBT-100 decreases the expression of immune checkpoint inhibitors in various cancer cell lines. (**A**) Surface expression of checkpoint inhibitors, B7-H3, OX-2, and PD-L1, was determined by flow cytometry in SJSA-1 osteosarcoma cells in the presence or absence of SBT-100, upon stimulation with IFN-γ (75 μg/mL). Fluorescent microscopy of PD-L1 expression in MDA-MB-231 cells without SBT-100 (**B**) and with SBT-100 (**C**). Confocal fluorescent microscopy of PD-L1 expression in MDA-MB-231 cells without SBT-100 (**D**) and with SBT-100 (**E**), both with the nuclei detection with DAPI. The scale bars in the lower-left corner of panels (**B**–**E**) represent 50 µm.

Next, we aimed to investigate the effect of SBT-100 on immune checkpoint inhibitors in cancer cells, such as PD-L1, a transcriptional gene target of STAT3. Checkpoint molecules are expressed on the surface of tumor cells and ultimately stifle the functioning of activated T cells, contributing to the immunosuppressive tumor microenvironment. Inflammatory cytokines such as IL-6 and IFN-γ drive the expression of checkpoint molecules such as PD-L1 (CD274), B7-H3 (CD276), and OX-2 (CD200)^23^. Culturing the human osteosarcoma cell line SJSA-1, with SBT-100 resulted in decreased cell surface expression of B7-H3, OX-2, and PD-L1 upon stimulation with IFN-γ as demonstrated by FACS analysis. B7-H3 decreased by approximately 3-fold, OX-2 decreased by approximately 4-fold, and PD-L1 decreased by approximately 4-fold (Figure 3A). We then analyzed MDA-MB-231 cells after 24 h of treatment with SBT-100 to examine potential changes in the level of PD-L1 protein synthesis within other cell types (Figure 3B–E). PD-L1 staining using IFA indicated lower membrane and intracellular protein levels mediated by SBT-100, consistent with published data on the expression of cellular PD-L1 localization. IFN-γ is known to induce PD-L1 expression and both the intracellular and membrane staining increased following treatment with IFN-γ (data not shown). We believe this is the first demonstration of downregulating checkpoint inhibitors at the gene expression level by an intracellular antibody.

**Figure 4:**
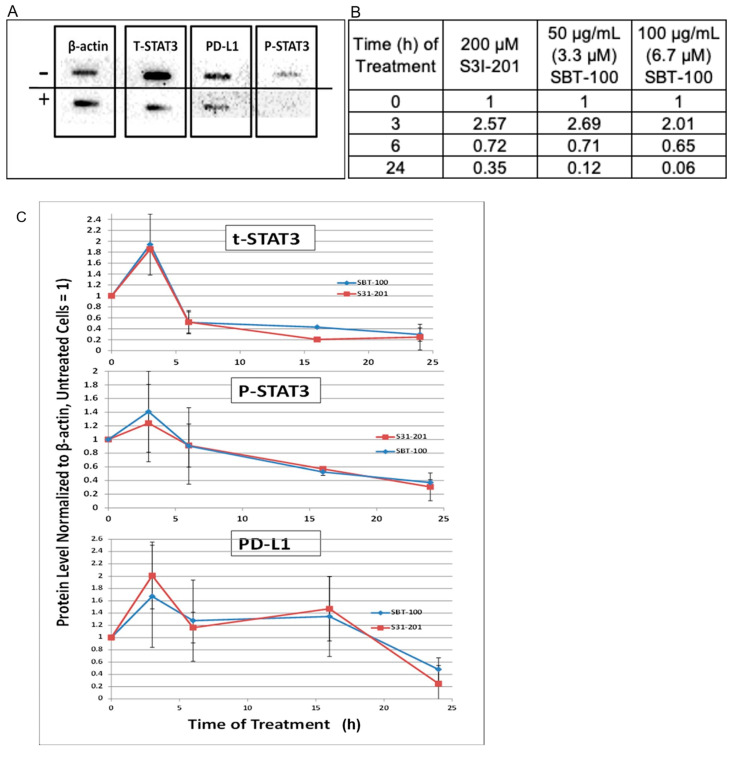
SBT-100 reduces pSTAT3, tSTAT3, and PD-L1 levels in total cell lysate. (**A**) Representative image of immunoblotting of MDA-MB-231 protein extracts for pSTAT3, tSTAT3, and PD-L1 with (+) and without (−) SBT-100 at 24 h. (**B**) Quantification of immunoblotting from MDA-MB-231 cells for pSTAT3, tSTAT3, and PD-L1 with and without SBT-100 treatment over a 24 h time course, using S31-201, a STAT3 inhibitor as a positive control. (**C**) Each data point represents the average ± the standard deviation of the β-actin normalized values from 3–5 independent experiments where MDA-MB-231 cells were treated with four different SBT-100 preparations whose final concentration varied between 50–100 μg/mL.

We then quantified the changes in the protein levels using the immunoblot technique (Figure 4A). Intracellular protein levels of total STAT3, pSTAT3, and PD-L1 were reduced in a time-dependent manner with SBT-100 treatment in MDA-MB-231 cells (Figure 4B,C). Replicate immunoblots were reacted with antibodies for each of the three proteins under investigation. Following chemiluminescent detection, the signal intensity of each protein in each extract was normalized to the intensity of the β-actin present in that sample. The levels in each treated sample were then compared to the level found in untreated cells (set to 1) to quantify the differences in the levels of tSTAT3, pSTAT3, and PD-L1 induced at multiple times post SBT-100 administration. S3I-201, a small molecule STAT3 phosphorylation inhibitor which has been shown to decrease levels of PD-L1, was used as a positive control. SBT-100 treatment reduced levels of tSTAT3, pSTAT3, and PD-L1, with the most significant reduction of all three proteins at 24 h. Thus, quantification confirmed the data observed by immunofluorescence. 

The nuclear portion of the staining with the commercially purchased tSTAT3 antibody and the pSTAT3 antibody were determined from the two-dimensional images by quantification of the green antibody-specific fluorescence that co-occurred with the nuclear (blue DAPI) staining. Following 6-h treatment with SBT-100, 41% of the original tSTAT3 staining and 70% of the original pSTAT3 staining remained in the nucleus. Of the tSTAT3 still present, none remained within the nucleus based on a three-dimensional projection of the corresponding Z-stacked images obtained by confocal microscopy. Any tSTAT3 that co-stained with DAPI in the two-dimensional images was present in the cytoplasm surrounding the nuclear membrane. The very low staining intensity of the pSTAT3 antibody in the IFA system makes this analysis impossible using that antibody. These data show that SBT-100, like S3I-201, decreased tSTAT3, pSTAT3, and PD-L1 over the course of the 24-h treatment. There appeared to be an early (3hour) increase in all three-protein species prior to a decrease. The reduction in tSTAT3 was evident at 6-h and preceded that of pSTAT3, while reduced PD-L1 levels were apparent 24-h after treatment with SBT-100. Taken together, these data support the hypothesis that the binding of STAT3 to SBT-100 decreases PD-L1 translation by decreasing levels of the pSTAT3 transcription factor.

### 2.4. Inhibition of IL-6 Induced STAT3 Nuclear Translocation and Suppression of STAT3 Mediated VEGF Production

**Figure 5:**
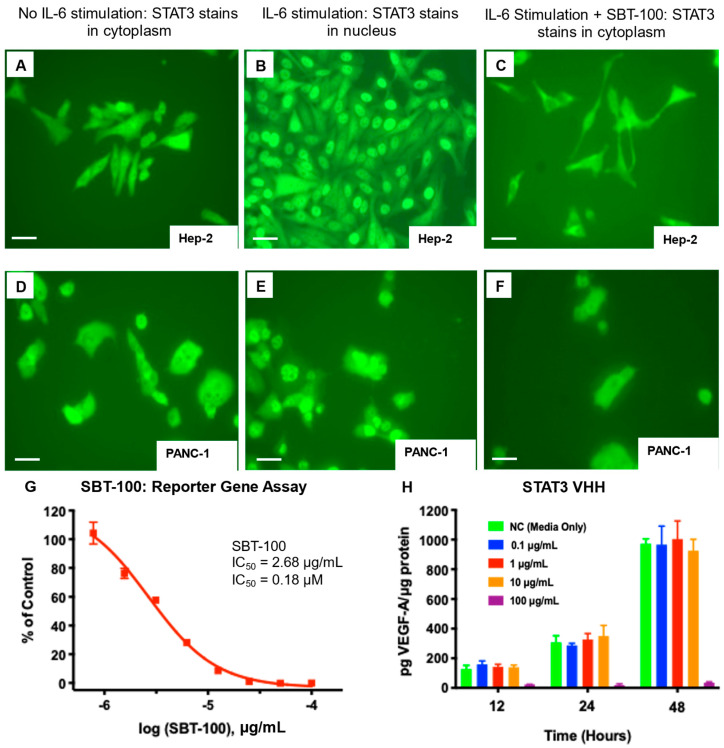
SBT-100 inhibits IL-6-induced nuclear translocation of pSTAT3 and VEGF production in various cell lines. Fluorescent microscopy of phosphorylated STAT3 in Hep-2 cells under normal conditions (**A**), upon IL-6 stimulation (**B**), and with both IL-6 stimulation and SBT-100 treatment (**C**). Fluorescent microscopy of phosphorylated STAT3 in Panc-1 cells under normal conditions (**D**), upon IL-6 stimulation (**E**), and with both IL-6 stimulation and SBT-100 treatment (**F**). The scale bars in the lower-left corner of panels (**A**–**F**) represent 50 µm. (**G**) STAT3 luciferase reporter assay in HEK 293T cells with SBT-100 treatment after IL-6 stimulation. (**H**) Measurement of VEGF levels by ELISA in retinal epithelial cells stimulated with IL-6 in the presence of increasing concentrations of SBT-100. Values are normalized to the total protein level. At 12, 24, and 48-h time points, SBT-100 at 100 μg/mL gave > 99% suppression of VEGF protein production (*p* < 0.0001). The scale bars in the lower-left corner of each panel represent 50 µm.

Since IL-6 plays a key role in modulating nuclear translocation of activated pSTAT3, we next sought to determine whether SBT-100 mediates its activity by inhibiting IL-6 activity. As shown in Figure 5A–F, IL-6 stimulation of HEp-2 and PANC-1 cells with SBT-100 inhibited nuclear translocation of pSTAT3, subsequently reducing the proliferation of HEp-2 and PANC-1 cells. Reporter cell assay was designed to measure luciferase expression from HEK 293 cells transfected with a construct linking the STAT3 promoter to the luciferase gene (Figure 5G). Using a luciferase reporter assay system in HEK 293 cells, STAT3 activity was measured in the presence of IL-6. Upon stimulation with IL-6, STAT3 dimers bind to the STAT3 promoter, inducing luciferase activity. The addition of SBT-100 abrogated this effect in a dose-dependent manner. Thus, the IL-6 effect on STAT3 activity was significantly inhibited by SBT-100. This assay showed that treatment with SBT-100 renders near 100% reduction in IL-6 induced pSTAT3 binding to its DNA promotor at a dose of 100 µg/mL, with an IC_50_ of 2.68 µg/mL (0.18 µM).

In cancer cells, STAT3 transcribes many genes necessary for the growth and survival of cancer, such as VEGF. To determine the ability of SBT-100 to inhibit VEGF production, we used retinal epithelial cells that produce VEGF in large quantities. Here we found that SBT-100 significantly (*p* < 0.0001) impedes VEGF production in retinal epithelial cells. At 12, 24, and 48-h time points, SBT-100 at 100 µg/mL gave > 99% suppression of VEGF protein production (Figure 5H). No cell toxicity was observed at any concentration tested in this experiment (data not shown). VEGF is a critical cytokine for the growth and survival of cancer cells, and it is well known that inhibition of VEGF function can significantly improve the overall survival of cancer patients (colorectal, lung, glioblastoma, kidney, cervical, and ovarian cancers) [23]. For instance, bevacizumab hinders the effect of VEGF by binding it in the extracellular space. Figure 5H demonstrates that SBT-100 penetrates the cell membrane and inhibits STAT3 function resulting in significantly decreased VEGF protein production. This is a completely unique way of inhibiting VEGF as compared to bevacizumab.

### 2.5. SBT-100 Inhibits Growth of Human Cancers with KRAS Mutations and Constitutive Expression of pSTAT3 In Vitro

Additionally, SBT-100 impaired growth in eleven human cell lines derived from a variety of cancers, including pancreatic cancers (PANC-1 and BxPC-3), TNBCs (MDA-MB-231, MDA-MB-468, MDA-MB-453), ER+PR+ breast cancer (MCF-7), HER-2+ amplified breast cancer (BT474), glioblastoma (U87), osteosarcoma (SJSA-1), fibrosarcoma (HT-1080), and metastatic, chemo-resistant prostate cancer (DU-145) (Table 2). The MDA-MB-231 cells have a KRAS(G13D) and PANC-1 cells have a KRAS(G12D) activating mutation. Most of these cancer cells have a constitutive expression of pSTAT3. These results demonstrate that SBT-100 possesses a wide spectrum of anti-cancer activity. This data suggests that SBT-100 has significant (*p* < 0.001) tumor cell inhibitory effects (85–93%) against human malignancies with constitutive pSTAT3 expression with or without an activating KRAS mutation.

### 2.6. Inhibition of KRAS GTPase Activity and Suppression of Downstream pERK Signaling

**Figure 6:**
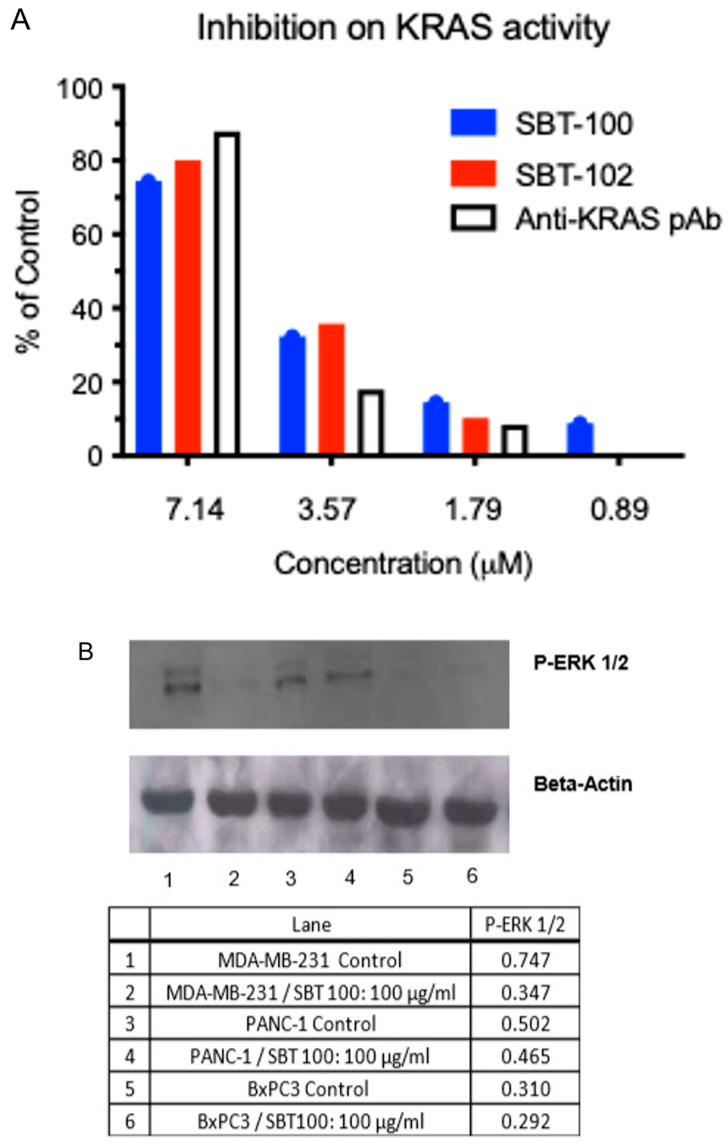
SBT-100 inhibits the GTPase activity of KRAS and stifles downstream ERK phosphorylation in KRAS mutant cancer cells. (**A**) Luminescence (RLU) was measured as a readout for KRAS GTPase activity. Reagents were incubated with either SBT-100, SBT-102, or anti-KRAS polyclonal antibody, and RLU were measured. (**B**) Western blot analysis for phosphorylated ERK1/2 in various KRAS mutant cancer cells with and without SBT-100 treatment. In Lane 1 (MDA-MB-231), Lane 3 (PANC-1), and Lane 5 (BxPC3) cancer cells were incubated with a vehicle only. In Lane 2 (MDA-MB-231), Lane 4 (PANC-1), and Lane 6 (BxPC3) cancer cells were incubated with 100 µg/mL of SBT-100 for 72 h.

Binding data demonstrates SBT-100 is bi-specific for KRAS and STAT3 as determined using these proteins and Lipoxygenase, while SBT-102 is mono-specific for human KRAS and its most common mutant KRAS(G12D) with nanomolar affinity but does not bind STAT3 (Table 1). We then demonstrated the biochemical effect of SBT-100 and SBT-102 on KRAS using GTPase activity. KRAS GTPase activity is inhibited by increasing doses of SBT-100 and SBT-102, demonstrating its inhibitory binding activity (Figure 6A). Here anti-KRAS polyclonal antibody was used as a positive control. Inhibition of KRAS GTPase activity by SBT-100, SBT-102, and anti-KRAS polyclonal antibody occurred in a dose-dependent manner, and the results between the three were comparable. Furthermore, downstream phosphorylation of ERK) is inhibited in both MDA-MD-231 and PANC-1 cancer cell lines harboring KRAS mutations (Figure 6B). By binding KRAS and inhibiting its GTPase activity, SBT-100 blocks the KRAS signaling pathway. We hypothesize cancer cells with greater basal STAT3 activity will trap SBT-100 within the cytoplasm to a greater extent, thereby enhancing SBT-100′s ability to inhibit KRAS. This is likely the explanation for the difference in pERK levels between these cancer cell lines. It has been demonstrated that MDA-MB-231 has much higher levels of STAT3 than PANC-1 [19]. Again, this may result in decreased time for SBT-100 to bind and inhibit KRAS. In addition, the literature shows that PANC-1 is KRAS-independent whereas other cancers with KRAS mutations are KRAS-dependent [24]. This may also explain the lower reductions in pERK levels in PANC-1 versus MDA-MB-231. Thus, SBT-100 has demonstrated binding to KRAS, subsequently, inhibiting of KRAS GTPase activity and suppression of downstream KRAS signaling by reducing pERK levels which resulted in the inhibition of the growth of cancer cells with activating KRAS mutations. 

### 2.7. Reduction in Tumor Volume In Vivo with SBT-100 in Tumor Bearing Mice 

**Figure 7:**
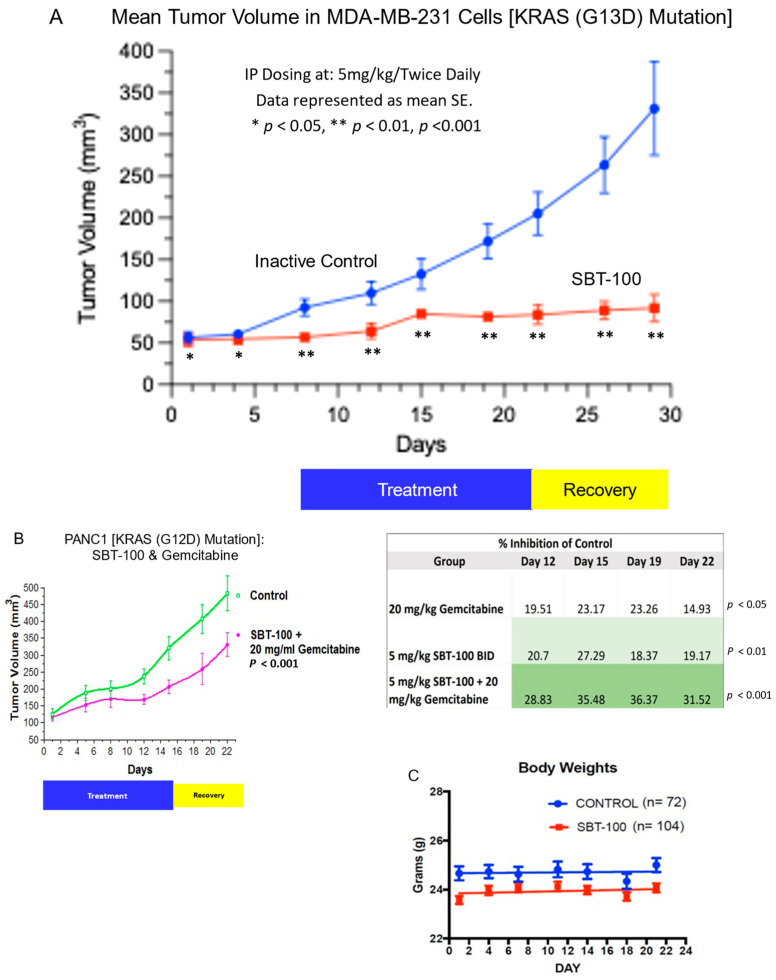
SBT-100 reduces tumor growth in mouse tumor xenograft models. (**A**) Athymic nude mice were injected with MDA-MB-231 cells subcutaneously in their right flanks. Once tumors were between 50–100 mm^3^, mice were treated with either saline vehicle or SBT-100 (5 mg/kg, BID) via intraperitoneal injection for 14 days until sacrifice. (**B**) Athymic nude mice were injected with Panc-1 cells subcutaneously in their right flanks. Once tumors were between 100 mm^3^–150 mm^3^, mice were treated with saline vehicle, gemcitabine (20 mg/kg, every third day), SBT-100 (100 mg/kg, BID), or combination gemcitabine and SBT-100, all via intraperitoneal injection for 14 days. After this 14-day period of treatment, there was a 7-day period of just observation. No weight loss or toxicity occurred in any of the treated mice. (**C**) In pooled data of mice weights from multiple studies. Mice treated with SBT-100 did not lose weight or show any signs of toxicity over the 3 weeks during these xenograft studies.

Finally, athymic mice were subcutaneously injected with a TNBC cell line (MDA-MB-231) or pancreatic cancer cells (PANC-1) and after approximately 3 weeks of growth, they were treated for 14 days with SBT-100 and allowed to recover for 7 days for observations of tumor volume. MDA-MB-231 tumors were between 50–100 mm^3^ prior to initiation of treatment (Figure 7A). These tumor-bearing mice were randomized into a control group which received PBS and a treatment group that received SBT-100. The treatment group demonstrated rapid suppression of tumor growth, and this was maintained even after the 14-day treatment period. During the 7-day post-treatment observation period, no rebound in tumor growth occurred, and all the mice survived treatment and maintained their normal weight. After the last dose of SBT-100, there was no significant growth of the MDA-MB-231 during the observed period.

PANC-1 tumors after approximately 3 weeks of growth were between 100–150 mm^3^ prior to treatment and then randomized into four groups: (1) control (PBS), (2) gemcitabine only, (3) SBT-100 only, and (4) gemcitabine with SBT-100 (Figure 7B,C). At the end of the 3-week study, the gemcitabine only group had 14.93% tumor growth suppression versus the control group. The SBT-100 only group had 19.17% tumor growth suppression versus the control group. Finally, the combination group of gemcitabine with SBT-100 gave 31.52% suppression versus the control group (*p* < 0.001). No treated mice died or had any weight loss with SBT-100. To determine the potential toxicity of SBT-100, we combined the weight data from all xenograft studies where the mice received SBT-100. During the 3-week xenograft studies, there was no significant weight loss in the groups receiving SBT-100 for treatment (Figure 7C). Reasons for the lack of observable toxicity may include the short serum half-life while biological effects lasting for up to 7-days which are reversible. In addition, the role of STAT3 in normal adult tissues is limited. Conditional ablation of STAT3 in adult mice has been shown to impact different systems to varying degrees without being lethal, unlike embryonic targeting that results in lethality [25,26]. It may also be possible that due to aberrant levels of intracellular STAT3 expression in cancer cells, the injected SBT-100 may be preferentially accumulating in the cancer lesions although we have not demonstrated the differential accumulation in this report.

**Figure 8:**
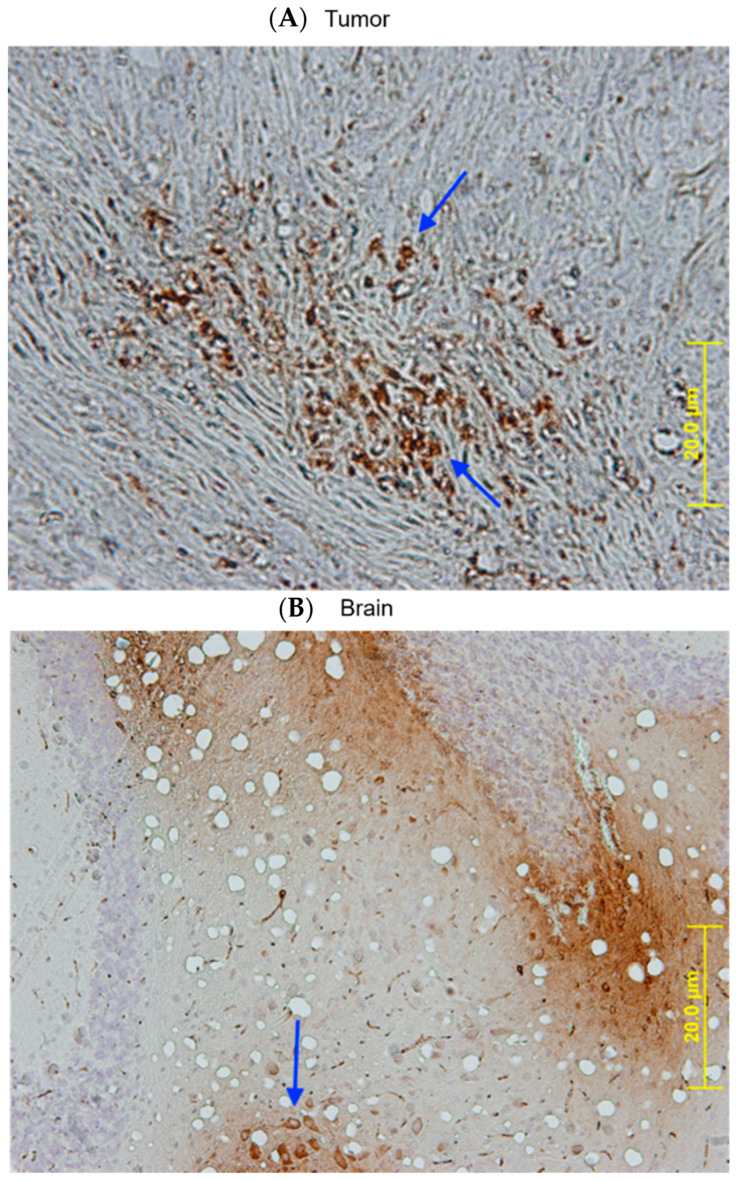
SBT-100 rapidly crosses the cell membrane of cancer cells and the blood-brain barrier in vivo. Athymic nude mice with large established MDA-MB-231 tumors (>200 mm^3^) were injected once IP with 5 mg/kg of SBT-100. Fifteen minutes later, these mice were sacrificed. The tumors and brains of these mice were then processed for staining. Immunohistochemistry analysis demonstrates the localization of SBT-100 inside cancer cells across the blood-brain barrier (BBB). (**A**) This representative section of the tumor mass shows brown intracellular staining within the TNBC cells as indicated by the arrows. These cells are surrounded by dense tumor stroma. (**B**) The arrow points to a collection of brown intracellular staining of neurons and glial cells within the brains of the tumor-bearing mice.

Another important finding in vivo is that SBT-100 crosses the blood-brain barrier (BBB) in vivo. Tumor-bearing mice injected with SBT-100 intraperitoneally (IP) were sacrificed 15 min later. Fluorescent microscopy revealed that SBT-100 localized within the cancer cells of the tumor and within the neurons and glial cells of the brain (Figure 8). It has been demonstrated that VHHs with basic pI (9.3 but not 7.7) could spontaneously cross BBB and enter astrocytes^30^. SBT-100 has a basic pI of 8.2 (calculated using Prot Pi online tool), suggesting a pI range of 8–10 may enable BBB crossing and cell penetration.

## 3. Discussion

Despite the short half-life of VHHs in serum, camelid VHHs are increasingly considered for clinical use due to their ability to target antigens residing in tissues that are poorly vascularized and not easily accessible. In addition, it is stable at room temperature and in reducing the cytoplasmic environment [19]. Here we have described a unique first-in-class VHH, SBT-100 with cell-penetrating capability, that can bind to two different non-homologous intracellular targets (STAT3 and KRAS) implicated in tumorigenesis. In our binding studies, SBT-100 binds STAT3 and KRAS but not 12-lipoxygenase. Indeed, a more exhaustive panel of proteins in the same binding study will help further determine if SBT-100 is truly bi-specific and not polyreactive. The serum half-life of SBT-100 is 1 h (data not shown) and this is consistent with the literature. If SBT-100 was polyreactive, then its serum half-life would likely be much longer because it would bind to random serum proteins with high affinity. We demonstrated that SBT-100 (1) crosses the cell membrane and binds to intracellular STAT3, (2) inhibits phosphorylation of STAT3, (3) decreases total STAT3, (4) blocks IL-6 mediated translocation of activated STAT3 into the nucleus, to prevent pSTAT3 dimers from binding to its target genes, (5) inhibits expression of VEGF, a key angiogenic factor and known modulator of tumor cells, (6) inhibits cell surface expression of checkpoint inhibitor PD-L1 on the tumor cell surface, which may improve antitumor immunity in immune-competent mice, (7) inhibits KRAS-GTPase activity and downstream ERK phosphorylation to inhibit cell growth, (8) exhibits wide-ranging anti-tumor cell growth in vitro against eleven human cancers, and (9) induces tumor (human cancers with activating KRAS mutations) regression in athymic xenograft mouse models for triple-negative breast cancer cell line (MDA-MB-231) and for pancreatic cancer (PANC-1), without any observable toxicity. The biological effects of SBT-100 are reversible, lasting at least 72 h in vitro and 7 days in vivo. SBT-100 also appears unique in its ability to penetrate BBB. The ability of SBT-100 to penetrate the cell membrane and bind intracellular KRAS and STAT3 translates into functional suppression of cancer growth and proliferation in vitro and in vivo. This was demonstrated with multiple human cancers to show the broad application of SBT-100 inhibiting human cancers.

SBT-100 rapidly crosses the cell membrane in vitro in less than six hours (Figure 1), and in vivo, it crosses the BBB in less than fifteen minutes (Figure 8). If SBT-100 is polyreactive, then it would likely bind cell surface proteins non-specifically and aggregate at the cell surface. This is not observed (Figure 1, Figure 2, Figure 3B–E and Figure 5A–F). It would then enter the cell via endocytosis and become part of the lysosomal pathway and become degraded, losing its efficacy. This was not observed. Upon entering the cell, SBT-100 binds non-covalently to KRAS and STAT3 with nanomolar affinity (Table 1). Unlike small molecule inhibitors which form irreversible covalent bonds [27], SBT-100 is less likely to create toxicity due to non-covalent reversible binding to KRAS and STAT3. Our hypothesis is that SBT-100 passes through all cells non-specifically and in adult mice and humans STAT3 is not actively produced in most tissues as found in the fibrosis literature (e.g., human skin and lung biopsies from healthy people reveal no STAT3). In these normal tissues where there is very little or no activated STAT3, SBT-100 simply passes in and out of these cells; however, in cancer cells where there is hyperexpression of pSTAT3, SBT-100, upon entering these cells, binds to the pSTAT3 with nanomolar affinity and becomes sequestered. Within 1 h, 50% of the SBT-100 has been excreted into the urine of the mice, so that any potential toxicity in some normal cells, which have tightly regulated pSTAT3 such as myeloid cells, is minimized. We have treated at IITRI close to 200 xenograft mice with SBT-100 for 14 consecutive days or 21 consecutive days and have had no deaths and no weight loss in any treated mice. At the NIH, our collaborators had a similar experience (35). We hope to begin GLP toxicology studies in rats and dogs. This will provide more rigorous data on the potential toxicity of SBT-100.

The blocking of the GTPase activity of KRAS (Figure 6A) and subsequent decreases in the levels of pERK1/2 (Figure 6B) inhibits the ability of the KRAS pathway to promote cell proliferation, survival, and escape apoptosis. Concurrently, SBT-100 also binds to STAT3 which causes inhibition of STAT3 phosphorylation (Figure 3, Figure 5 and Figure 6), the inability of STAT3 to translocate into the nucleus, and prevent STAT3 from binding to its DNA promotor (Figure 5G). A powerful example of SBT-100′s inhibitory and anti-inflammatory ability is also demonstrated by its ability to block the effect of IL-6 on cancer cells and normal cells in vitro (Figure 5) by preventing STAT3 from transcribing target genes in the nucleus such as VEGF and PD-L1. We are aware of the fact that SBT-100 may bind both molecules or preferentially to one depending on the local concentrations of KRAS and STAT3. We do not know the structural aspects of SBT-100 binding to these proteins. Such non-homologous protein cross-reactivities have been observed among allergens binding to IgE antibodies [28].

At this time, we can only speculate as to how SBT-100 penetrates live cells to cause therapeutic effects. Herce and Garcia proposed that a direct translocation of cationic proteins by positively charged amino acids interacting with negatively charged phosphate groups of the cell membrane form transient pores using HIV-1 TAT cell-penetrating peptide (CPP) [29]. Li et al. [30] speculated that a similar phenomenon may be at work for Glial fibrillar acidic protein (GFAP) nanobody E9, in the crossing of BBB and intracellular penetration and further claimed that their monomeric VHH not only acted as a CPP but also specifically bound to intracellular antigen both in vitro and in vivo [30]. They also observed an association between size and cellular penetration. While a smaller quantity of VHHs crossed BBB in vitro, monomer uptake was greater than that of dimer VHH (7.8% vs. 4.3%). Based on the E9 VHH sequence (sdAb-DB Accession Number: sdAb_0207_Vp) published on the single-domain antibody database (http://www.sdab-db.ca (accessed on 8 August 2020)) [31], the sequence-based theoretical pI for E9 is 8.7 (their experimentally calibrated pI was 9.4) with a net charge of +2.2 at physiological pH of 7.4 whereas for HIV-1 TAT peptide, a prototypical widely used CPP, the pI is 12.5 with a charge of +6.2 and for SBT-100 the pI is 8.2 with a net charge of +2.3 at pH 7.4, suggesting biochemical similarities may exist between cell-penetrating camelid nanobodies. This also suggests a superior capability for TAT peptides to transport molecules across the cell membrane compared to that of camelid nanobodies.

VEGF plays a critical role in tumor growth and metastasis by producing the development of new blood vessels. SBT-100 significantly reduces the production of VEGF by retinal epithelial cells in vitro as rapidly as 12 h, and the biological effect of a single administration lasts at least 48 h (Figure 5H). This suggests SBT-100 may reduce anti-tumor effects in cancer and may reduce blindness in neovascular conditions such as age-related macular degeneration (AMD). In an in vivo model for blindness, SBT-100 has been shown to give a significant improvement in vision (data not shown). Other gene targets for STAT3 are PD-1 and PD-L1 [32,33]. Here using IFA, we demonstrate that SBT-100 decreases PD-L1 expression on TNBC (MDA-MB-231) within 24 h (Figure 3). Similar results were obtained for osteosarcoma (SJSA-1) where FACS analysis showed SBT-100 decreased PD-L1 expression within 48 h (Figure 3). We believe this represents a new approach to immunotherapy by downregulating a checkpoint inhibitor gene. This strategy of using SBT-100 may decrease the number of PD-L1 and possibly PD-1 molecules via decreasing STAT3 availability so there are fewer cell surface targets for nivolumab and pembrolizumab to block. This then may augment the checkpoint inhibitor response or enable reductions in the dosage of checkpoint inhibitors. Since STAT3 is a pro-inflammatory mediator, STAT3 inhibition by SBT-100 may also decrease some of the inflammatory complications associated with checkpoint inhibitor therapy such as pneumonitis [34] and severe COVID-19 pathology.

The therapeutic potential for SBT-100 in vivo has been demonstrated here as monotherapy, we have confirmed the efficacy in the form of tumor regression in athymic nude mouse xenograft with TNBC tumors with KRAS(G13D) mutation (at least 50–100 mm^3^). The therapeutic effect of SBT-100 persisted for at least seven days after the last dose. Similarly, SBT-100 augmented the suppression of tumor growth when combined with gemcitabine. PANC-1 is known to be difficult to treat malignancy since it is KRAS-independent [24]. These experiments suggest that SBT-100 alone or in combination with other chemotherapeutic agents cause significant tumor growth suppression in vivo.

While cell penetration by SBT-100 and anti-cancer clinical activities are exciting, the therapeutic effects of SBT-100 on ocular inflammatory diseases such as uveitis have also been validated by collaborators at the National Eye Institute at the National Institutes of Health. Briefly, Mbanefo et al. [35] used an experimental autoimmune uveitis (EAU) model which is the model for human uveitis to demonstrate that SBT-100 was efficacious and safe in treating the autoimmune attack of the retina. Their results demonstrated that SBT-100 crosses the blood neuroretina barrier and downregulates the generation of pSTAT3, decreases autoimmune Th1 and Th17 cells significantly, and reduces serum levels of IL-17A, IFN-γ, GM-CSF, and IL1-α. The group treated with SBT-100 showed minimal damage to the retina, preservation of the optic disc, normal histology, minimal infiltration of immune cells around the retina, and preservation of electrical conduction by the retina when compared to the control group.

Unlike conventional monoclonal antibodies which mostly recognize flat and convex antigenic surfaces, the CDR3 loop of VHHs is longer than conventional VH, which allows its extended paratope binding to non-conventional epitopes such as concave-shaped protein clefts such as enzyme active sites [22]. Although structural data on binding has yet to be carried out, it is theoretically feasible that SBT-100 binds to a shallow cleft described for KRAS by electrostatic interactions [13], or yet an undetermined cleft in KRAS. Thus, our SBT-100 appears unique in its ability to penetrate the cell and blood-brain barrier (BBB) along with its binding to STAT3 and KRAS. One potential concern is whether SBT-100 is immunogenic. As with any foreign protein, the risk of immunogenicity exists. Our 14-day treatment with SBT-100 is too short to detect any loss of efficacy due to immunogenicity. A full 28-day GLP toxicology study with immunogenicity testing will help give better information about this question. Fortunately, camelid VHHs, like SBT-100, are 90% or greater in homology with human VH proteins which should reduce the risk for immunogenicity. Since SBT-100 has a very short serum half-life and stays within the intracellular space of cancer cells, these two facts may further reduce its immunogenic potential. In addition, intracellular delivery of SBT-100 may induce anti-SBT-100 immunity in immunocompetent hosts. This may initially help promote anti-tumor immunity but in time this immunogenicity may reduce SBT-100′s efficacy. Further studies to determine this are warranted.

Mechanistically, the inhibitory action of SBT-100 likely involves steric hindrance created after binding of this agent to STAT3 and KRAS. Identification of epitope(s) on STAT3 and KRAS as seen by SBT-100, in vitro competitive binding, and structural aspects of binding between SBT-100 and its targets still need to be elucidated. The dual inhibitory property of SBT-100 may help to reduce the chance of cancer resistance from developing when used. It has been described that there exists an inverse correlation between STAT3 and MEK signaling which mediates resistance to RAS pathway inhibition in pancreatic cancer [36]. Thus, SBT-100 is a “First-in-Class” anticancer agent for targeting STAT3 and KRAS simultaneously in cancers and provides proof of concept for this novel approach to treating cancer and other diseases by focusing on aberrant intracellular targets. Further investigation into pharmacodynamic and pharmacokinetic studies are warranted. 

## 4. Material and Methods

### 4.1. SBT-100 Development

Recombinant full-length human STAT3 with a GST tag fused to its N-terminal (STAT3-1496H) was provided by Creative BioMart (Shirley, NY, USA). Briefly, a camel (*Camelus bactrianus*) was used for immunization with the recombinant human STAT3. Generating SBT-100 VHH: A Camelid was immunized with the relevant antigen. After the immunization period, peripheral white blood cells (PWBC) were collected, and a phage display library was created to look for the VHH of interest. Once the panning process was completed, VHHs were identified by their binding affinity to STAT3 and KRAS. The process used is described in greater detail in our previous publication [37]. The final endotoxin levels were <1 EU/mg.

### 4.2. Cell Lines and Cell Culture

Cell lines: PANC-1, BxPC3, MDA-MB-231, MDA-MB-468, MDA-MB-453, MCF-7, BT474, U87, SJSA-1, HT-1080, HEp2, DU-145 and retinal epithelial cells (ARPE-19) were all obtained from American Type Culture Collection (ATCC) (Manassas, VA, USA). All cells were grown at 37 °C in 5% CO_2_ in either DMEM or RPMI media with or without fetal bovine serum.

### 4.3. Immunofluorescence and Immunohistochemical Staining

Standard procedures were used for immunohistochemistry and immunofluorescence assay (IFA) staining. Primary antibodies for IFA were: Anti-t-STAT3: (Cat No. 30835, Cell Signaling Technology, Danvers, MA, USA), Anti-p-STAT3: (Cat No. 9145, Cell Signaling Technology), Anti-PD-L1: (Cat No. 13684, Cell Signaling Technology), Anti-VHH Antibody: (Cat. No. 200-401-GM6S, Rockland, Rockland, MA, USA), Alexa Fluor 488-Anti-rabbit IgG: (Jackson ImmunoResearch, West Grove, PA, USA, Cat. No. 711-545-152), rabbit polyclonal anti-GST: (Thermo Fisher #71-7500, Waltham, MA, USA). The blocking solution, 1° and 2° antibody diluent were 1% BP: (1% BSA in PBS). All cell incubations were at 37 °C in a 5% CO_2_ incubator in media. 4500 cells/well were seeded in chamber slides and allowed to adhere overnight. Cells were treated with SBT-100 at various timepoints and then fixed in 100% methanol at −20 °C. For IFA staining, slides were blocked for ≥30 min with 1% BP, the blocking agent removed, and 1° antibodies were added at the following dilutions prior to overnight incubation at 5 °C: anti-VHH = 1:500, anti-t-STAT3 = 1:300, anti-p-STAT3 = 1:125, anti-PD-L1 = 1:300. Wells were washed with PBS and incubated with Alexa Flour labeled anti-rabbit IgG conjugate (1:300) for ≥1 h prior to washing with PBS, slides were cover slipped and examined by fluorescent microscopy. For DAPI staining, wells were incubated for 7min with 0.143 mM DAPI and washed with PBS prior to the application of a coverslip. Traditional fluorescence microscopy was performed using the Nikon 80i microscope and the appropriate wavelength filter. Images were captured using the attached Spot RT3 camera (Cat. No. WS-RT2540-0484, Spot Market Webstore, Sterling Heights, MI, USA, https://webstore.diaginc.com/) model 25.4, 2Mp slider and the associated Spot 5.1 software.

### 4.4. Confocal Microscopy

The confocal images were obtained using the Leica TCS SP8 confocal microscope with the expert assistance of Wade Sigurdson, Ph.D., Director of the Confocal Microscopy and Flow Cytometry Facility at the University at Buffalo, Buffalo, NY, USA. Images were quantified using Fiji software.

### 4.5. IHC Staining for Intra-Tumor and BBB Methodology

Athymic nude mice (n = 3) with established MDA-MB-231 tumors were injected IP with SBT-100 (1 mg/kg). Fifteen minutes later the mice were sacrificed, and their brains and tumors were harvested. The tissues were placed into 10% formalin for 24 h, and then transferred to 70% ethanol. These tissues were cut into sections with a dermatome (AML Laboratories, Baltimore, MD, USA). Goat anti-Ilama conjugated (Bethyl Laboratories, Cat. # A160-100P, Montgomery, TX, USA) secondary antibody (1:10,000) was incubated with these tissue sections for 10 min at room temperature, washed twice for 3 min with PBS-Tween 20. Incubation in streptavidin/peroxidase complex of the tissue sections was done for 5 min at room temperature, and then washed for 5 min with PBS. Next, the tissue sections were incubated with peroxidase substrate solution (AEC) for 15 min, washed in tap water for 5 min, counterstained with Hematoxylin QS (one drop on each section), and incubated for 30 s. Tissue sections were then rinsed with tap water until the water became colorless. The sections were mounted in aqueous mounting media, and 15 min later these slides were viewed on the Olympus BX51 Fluorescence Microscope.

### 4.6. Western Blot (Slot Blot)

Standard procedures were used for immunoblotting. Primary antibodies were: Anti-β-actin: (Cat. No. 4970, Cell Signaling Technology), Anti-t-STAT3: (Cat. No. 30835, Cell Signaling Technology) Anti-p-STAT3: (Cat. No. 9145, Cell Signaling Technology), Anti-PD-L1: (Cat. No. 13684, Cell Signaling Technology), HRP-Anti-rabbit IgG: (Cat. No. 711-035-152, Jackson ImmunoResearch). Blocking buffer, primary, and secondary antibody diluent 5% BT: (5% BSA in TBS) TBS (Tris Buffered Saline25 mM Tris, 150mM NaCl, pH 7.5) TBST (TBS + 0.1% Tween-20). Briefly, 2 × 10^5^ MDA-MB-231 cells/well were seeded in each well of a 6-well plate and allowed to adhere overnight at 37 °C in a 5% CO_2_ incubator in media. Media was removed and SBT-100 in media was added. Following incubation for the indicated times, media was removed, the adherent monolayer of cells was washed with ice-cold PBS and then lysis of the cells was performed by scraping the cells in TBS + 0.05% SDS with added EDTA, protease inhibitors, and phosphatase inhibitors. At later times of treatment where non-adherent cells were apparent, these cells were collected by centrifugation, lysis was performed, and combined with the lysate of the adherent cell population.

### 4.7. Quantification of Western Blot

Protein concentration of each fraction was determined using the BCA protein assay (Cat No. 23227, ThermoFisher Scientific). Equal amounts of protein from each of the cell fractions (typically ~10 μg/slot) were diluted to 200 μL/slot with TBS and loaded via a slot blot apparatus onto a PVDF membrane that had been previously activated in 100% methanol and then equilibrated in TBS. Blots were blocked for ≥1 h in 5% BT and then incubated at 5 °C overnight in the following dilutions of antibodies: anti-β-actin 1:1000, anti-t-STAT3 1:1000, anti-p-STAT31:750, anti-PD-L1 1:1000. Blots were washed three times with TBST, briefly equilibrated into TBS and then incubated with HRP-anti-rabbit IgG (1:5000) for ≥1 h prior. The washing step was repeated and then the blots were incubated with the chemiluminescent HRP substrate according to the manufacturer’s protocol. The reactions were visualized using a chemi-imager. Quantification was performed using the ImageJ software contained within the Fiji image processing package.

### 4.8. IL-6 Stimulation and Inhibition of pSTAT3 Nuclear Translocation

The cells (HEp-2 and PANC-1) were grown on 4 Permanox chambers slides. SBT-100 antibody was added overnight (1 to 10 dilutions in media) and the slides were kept at 37 °C. No SBT-100 antibody was added to the negative control samples. The following day, cells were stimulated with IL-6 (Peprotech, Cranbury, NJ, USA, 100 ng/mL) for 15 min. After stimulation, the chamber slides were immediately fixed in ice-cold 100% methanol for 10 min at 20°C. The slides were dried and proceeded with the previously mentioned IFA steps. Slides were blocked with 3% BSA in PBS at room temperature for 1 h, then the primary antibody, STAT3 (124H6, Cell Signaling Technology) overnight at 4 °C. The secondary antibody anti-mouse IgG (H and L), Alexa Fluor 488 (Cell Signaling Technology) was added for 1 h at room temperature. Lastly, the chamber slides were washed and mounted with our mounting media and viewed in the Nikon Fluorescence microscope.

### 4.9. Promega Dual Luciferase Reporter Assay System (GTPase-Glo™ Assay)

In this assay, we utilized a HEK 293 IL-6 STAT3 reporter cell line (Cat. No. E1910 Promega, Madison, WI, USA) to measure STAT3 transcriptional activity. In this cell line, induction with 40 ng/mL of IL-6, activates STAT3 transcription factors to drive luciferase reporter expression which can then be measured on a standard luminometer. We incubated 10^5^ cells/well with SBT-100 antibody or no antibody for 48 h. An 8-point, 2-fold titration, starting at 100 μg/mL was made to test the IC_50_ values. IL-6 was added during the last 18 h of incubation. All-time points are clocked from the addition of SBT-100 inhibitor. At 48 h, cells underwent lysis, and luminescence measurements were made using a BMG Labtech microplate reader. Results are expressed as a percent of control wells (cells + IL-6).

### 4.10. Human VEGF-A ELISA Assay

The human VEGF-A ELISA (Cat No. EHVEGF-AA, ThermoFisher Scientific) assay was modified to be performed in a 96-well plate format from a 24-well format. ARPE-19 cells were serum-starved in 10% FBS in DMEM and incubated overnight. The media was then replaced with media containing SBT-100 at 100, 10, 1, or 0.1 μg/mL, Anti-EMP2 antibody (Cat. No. ab174699, Abcam, Waltham, MA, USA), or just media and incubated for 12, 24, or 48 h. At the appropriate time point, the supernatant was removed and stored at −65 °C. Cells were lysed using RIPA lysis buffer (Cat No. 89900, ThermoFisher Scientific) and the protein content of the cell lysate was measured with a BCA assay (Cat No. 23227, ThermoFisher Scientific). VEGF-A was measured utilizing the Human VEGF-A ELISA Kit (Cat No. EHVEGF-AA, ThermoFisher Scientific) in accordance with the kit instructions. Experimental statistical analysis by ANOVA with Dunnett Multiple Comparisons Test using the negative control as the control column was performed. Statistical analysis by ANOVA with Dunnett Multiple Comparison Test using the negative control as the control was performed.

### 4.11. Flow Cytometry Analysis

SJSA-1 cells were incubated for 24 h with 50ng/mL recombinant human IFN-γ (Cat No. 300-02, Peprotech), then the media was replaced with media containing 50 μg/mL of SBT-100 and incubated for 48 h. Cells were then harvested and stained with the following antibodies: CD276 (Clone MIH-42, Biolegend Cat. No. 351005, San Diego, CA, USA), CD274 (Clone 29E.2A3, Biolegend Cat. No. 329713), and CD200 (Clone OX-104, Biolegend Cat. No. 329217). Upon staining, samples were run through a BD Celesta Flow Cytometer, and data were analyzed using FlowJo version 10.2 software (BD Biosciences, Ashland, OR, USA).

### 4.12. MTT Assay

For these experiments, cancer cells were grown until they reached a confluency of 90%. Cells were washed, trypsinized, and counted using a Coulter Counter (Beckman, Brea, CA, USA). The proliferation studies were carried out using the 3-[4,5-dimethylthialolyl]-2,5-diphenyl-tetrazolium bromide (MTT) assay (Roche Diagnostics Corporation, Cat. No. 11465007001, sold by Sigma-Aldrich, St. Louis, MO, USA). For this, cells were seeded in a 96-well plate at a density of 5 × 10^3.^ Cells were allowed to adhere for 24 h and treated at the appropriate concentrations (serial dilutions beginning at 100 μg/mL) as described in Table 2. On day 3, 10 μL of MTT reagent (0.5 mg/mL) was added to each well as indicated by the manufacturer. After a 4-h incubation period, 100 μL of solubilization solution was added and the plate was placed in the incubator overnight. All the plates were read at 570 nm wavelength using the Biotek plate reader (Winooski, VT, USA). All data were analyzed using GraphPad InStat3 (GraphPad Software, Inc., La Jolla, CA, USA). Treatment groups were compared with a vehicle control group using one-way ANOVA. If a significant difference (*p* < 0.05) was observed, then the Tukey-Kramer multiple comparison test was conducted.

### 4.13. Measurement of KRAS Inhibition Activity in an Enzymatic Assay

The GTPase activity of KRAS converts GTP to GDP. A GTPase-Glo reagent kit (Kit No., V7681, Promega, Madison WI, USA) is designed to measure this activity. The Glo reagent converts unhydrolyzed GTP to ATP and yields a luminescent signal. When KRAS activity is inhibited, GTP remains unhydrolyzed and a high Luminescent signal is expected. If KRAS is not inhibited, then the GTP is converted to GDP and a low signal is observed. Luminescence was measured using a PHERAstar plate reader (BMG Labtech, Ortenberg, Germany). We tested the activity of mature, active KRAS (Cat. No. R106-310H, SignalChem, Richmond, BC, Canada) supplied in the manufacturer’s buffer against the presence of a dilution series of inhibitors. The commercial KRAS GTPase activity was titrated in Promega GTPase/GAP buffer and in SBT-100 buffer in presence of several inhibitors. The inhibition and the effect of buffer conditions on the GTPase activity of KRAS were compared.

### 4.14. Animals

All animals were housed under pathogen-free conditions and experiments were performed in accordance with the Illinois Institute of Technology Research Institute (IITRI) Animal Use and Care Committee (IACUC) which is accredited by the Association for Assessment and Accreditation of Laboratory Animal Care International (AAALAC). Athymic nude-Foxn1nu female mice aged 5 to 6 weeks were purchased from ENVIGO Laboratories (Indianapolis, IN, USA). Animals were quarantined for one week and housed five mice per cage, with a 12-h light-dark cycle, at 20–26 °C, and relative humidity of 50%. Drinking water and diet (PicoLab Rodent Diet 20 Irradiated consisting of 20% crude protein, 4.5% crude fat, and 6.0% crude fiber) were supplied to the animal’s ad libitum.

### 4.15. Murine Xenograft Models

Tumor cells in passage five were used for the implantation and were harvested during log-phase growth. PANC-1 cells or MDA-MB-231 cells at a concentration of 5 × 10^6^ cells per 100 µL of media were injected subcutaneously into the right flank. Tumor measurements were initiated as soon as the tumors were palpable. Thereafter, tumors were measured twice weekly. Tumors were measured in two dimensions using calipers and volume was calculated using the formula: Tumor volume (mm^3^) = (w^2^ × l)/2; where w = width and l = length in mm of a tumor. Animals were randomized using the stratified random sampling algorithm when tumors reached a size range of 79–172 mm^3^ for PANC-1 tumors and 55–150 mm^3^ for the MDA-MB-231 tumors. Treatment of the animals with SBT-100 or vehicle-injected via intraperitoneal route was initiated the day following randomization referred to as day 1. Each group had at least four mice and was repeated on three separate occasions. Study Log Study Director Animal Study Management Software (San Francisco, CA, USA) was used to randomize animals, collect data (e.g., dosing, body weights, tumor measurements, clinical observations), and conduct data statistical analyses.

### 4.16. Statistics

The statistical methods used have been described in the individual sections above. Flow Cytometer data were analyzed using FlowJo v.10.2 Software. Graph Pad Prism v.9.0.1. All statistical analyses are two sided. Western blot analysis quantification was performed using the ImageJ software contained within the Fiji image processing package. ELISA assay statistical analysis by ANOVA with Dunnett Multiple Comparison Test using the negative control as the control was performed. MTT assay treatment groups were compared with a vehicle control group using one-way ANOVA. If a significant difference (*p* < 0.05) was observed, then the Tukey-Kramer multiple comparison test was conducted. In all the mice xenograft studies, Study Log Study Director Animal Study Management Software (San Francisco, CA, USA) was used to conduct data statistical analyses.

## Figures and Tables

**Table 1:** Binding affinity of SBT-100 for human STAT3 and KRAS determined using Biacore 3000.

		Human STAT3 Protein	Human KRAS Wildtype Protein	Human KRAS (G12D) Mutant
1	SBT-100 (Anti-KRAS/ Anti-STAT3 VHH)	2.24 × 10^−8^ M	4.20 × 10^−9^ M	1.50 × 10^−8^ M
2	SBT-102 (Anti-KRAS VHH)	No Binding Observed	3.22 × 10^−9^ M	1.48 × 10^−7^ M

**Table 2:** SBT-100 inhibited in vitro growth of eleven tumor cell lines. PANC-1 has a KRAS(G12D) mutation and MDA-MB-231 has a KRAS(G13D) mutation.

Cancer	Human Cancer Cell Line	% Inhibition in 3 Days with SBT-100 (100 μg/mL or 6.7 μM)	IC50 (μM)
Pancreatic	PANC-1	85% (*p* < 0.01)	0.38
Pancreatic	Bx-PC3	90% (*p* < 0.01)	2.34
TNBC	MDA-MB-231	89% (*p* < 0.01)	1.21
TNBC	MDA-MB-468	85% (*p* < 0.01)	0.83
TNBC	MDA-MB-453	64% (*p* < 0.01)	1.19
Breast	MCF-7	93% (*p* < 0.01)	0.99
Breast	BT474	93% (*p* < 0.01)	1.68
Glioblastoma	U87	62% (*p* < 0.01)	4.32
Osteosarcoma	SJSA	83% (*p* < 0.01)	3.39
Fibrosarcoma	HT-1080	86% (*p* < 0.01)	2.13
Prostrate	DU-145	92% (*p* < 0.01)	1.14

## Data Availability

Not applicable.
